# Protective effects of fermented rice extract on ulcerative colitis induced by dextran sodium sulfate in mice

**DOI:** 10.1002/fsn3.1460

**Published:** 2020-02-14

**Authors:** Won‐Seok Oh, Jae‐Chul Jung, Yong‐Min Choi, Ju‐Young Mun, Sae‐Kwang Ku, Chang‐Hyun Song

**Affiliations:** ^1^ Department of Veterinary Internal Medicine College of Veterinary Medicine Kyungpook National University Daegu Korea; ^2^ Life Science Research Institute NOVAREX CO., Ltd. Cheongju Korea; ^3^ Department of Anatomy and Histology College of Korean Medicine Daegu Haany University Gyeongsan Korea; ^4^ Research Center for Herbal Convergence on Liver Disease Gyeongsan Korea

**Keywords:** anti‐inflammation, antioxidant, inflammatory bowel disease, intestinal barrier, microbiota

## Abstract

Ulcerative colitis (UC) is a chronic inflammatory bowel disease (IBD), characterized by the gut mucosal ulceration. Growing evidence indicates that dysregulation of immune response to the commensal microbiota involves the pathogenesis of IBD. Previous studies have demonstrated the favorable probiotic effects of fermented rice extracts through triple fermentation with *Saccharomyces cerevisiae* and *Weissella cibaria* (FRe). Thus, the therapeutic potential of FRe for UC was examined. Dextran sodium sulfate UC mice model was orally administered distilled water as a control, sulfasalazine, or FRe at 300, 200, and 100 mg/kg, once a day for a week. The UC control exhibited body weight loss, bloody stools, and colonic shortening. However, the FRe, especially at 300 mg/kg, led to a reduction in weight loss, disease activity index scores, and colon weight, and an increase in colorectal length. The histopathological analyses revealed mild changes involved in the colonic crypt and mucosal damages in the FRe groups, along with inhibited inflammation. Indeed, the FRe reduced neutrophil infiltration and production of proinflammatory cytokines (i.e., tumor necrosis factor‐α, interleukin‐6/‐8). This was accompanied by the down‐regulation of nuclear factor‐kappa B. The gene expression responsible for the intestinal barrier integrity (i.e., *Zonna occludens‐1*/*‐2*, *Claudin‐1*, *Occludin*, *Mucin‐1*/*‐2*) was up‐regulated in the FRe groups. In addition, the FRe reduced lipid peroxidation and enhanced antioxidant activity. Interestingly, the microbiota dysbiosis was attenuated in the FRe groups, and the number of beneficial bacteria, *Lactobacilli* and *Bifidobacteria,* was increased. It suggests that the FRe potently ameliorate UC as a functional food.

## Graphical abstract

Oral treat of FRe ameliorated the UC symptoms by (a) improved dysbiosis as prebiotics, (b) NF‐κB‐mediated anti‐inflammation, (c) enhanced antioxidant activity, (d) restored mucus layer and the tight junction, (e) reduced immunity of CD4+ and CD8+ T cell, and (f) increased serum IgG but decreased IgA.

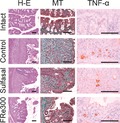

## 1 INTRODUCTION

Inflammatory bowel diseases (IBDs) include two main types of ulcerative colitis (UC) and Crohn's disease, which is characterized by chronic and recurrent inflammation with mucosal ulceration in the gut. (Hanauer, 2006). The disease symptoms are body weight loss, abdominal pain, diarrhea, and fatigue. Although the etiology remains uncertain, the pathogenesis involves several factors, such as the environmental factors affecting genetically susceptible individuals, excessive oxidative stress during inflammatory progression, epithelial barrier disruption, and abnormal immune reactions to the gut microflora (Fiocchi, 1998; Matsuoka & Kanai, 2015; Piechota‐Polanczyk & Fichna, 2014). IBD has long been regarded as a public health problem in Western countries. However, the incidence has been increasing in Asian countries with Western diets, excessive sugar intake, and low‐fiber consumption (Ruemmele, 2016). The major medications are focused on the control of inflammation and immunomodulation, and they include sulfasalazaine, prednisone, azathioprine, and antibodies against tumor necrosis factor (TNF)‐α and integrin (Hanauer, 2006). However, the drugs increase the risk of infectious complications and unexpected side effects, such as allergies and lymphoma. Thus, feasible long‐lasting and safe therapeutic treatments are urgently needed.

The gastrointestinal tract contains commensal microbiota, and the corresponding immune responses are normally down‐regulated to preserve homeostasis (Belkaid & Hand, 2014). Growing evidence indicates that the dysregulated immune response to the microbiota leads to the inflammatory process and eventually intestinal lesions in IBD (Belkaid & Hand, 2014; Filipescu et al., 2018). Indeed, the dysbiosis has been observed in IBD patients (Chassaing & Darfeuille‐Michaud, 2011); however, germ‐free animals remain the disease free (Sartor, 2008). UC is triggered predominantly by the epithelial invasion of the intestinal microbiota due to the loss of the epithelial layer integrity (Matsuoka & Kanai, 2015). This leads to the overproduction of proinflammatory cytokines (i.e., TNF‐α, interleukin [IL]‐1β and IL‐6) and colonic ulceration and damage. Thus, the potential efficacy of probiotics or prebiotics for the treatment of UC has engendered interest (Laurell & Sjoberg, 2017). They are considered as safe food constituents involved in the regulation of the microbita balance and intestinal function (Vanderhoof & Young, 1998), and selective growth‐stimulation of the beneficial microbiota (Komiyama et al., 2011).

Humans may have been unconsciously ingesting beneficial microbes through fermented foods as a main carrier to deliver probiotics for a long time (Linares et al., 2017). The epidemiological data have shown positive correlation between the occurrence of IBD in Japan and decreases of rice consumption (Asakura, Suzuki, Kitahora, & Morizane, 2008). Fermented rice extracts have been reported to have various pharmacological properties including hypoglycemic (Lu et al., 2010), antitumor (Ho et al., 2010), antistress, and antifatigue effects (Kim, Yu, Kang, & Suh, 2002). We previously developed fermented rice extracts through triple fermentation with *Saccharomyces cerevisiae* and *Weissella cibaria* (FRe), which contains galacto‐oligosaccharides as prebiotics. The FRe has shown favorable laxative effects in normal conditions (Choi et al., 2014) or constipation animal model (Choi et al., 2014), and it has no acute toxicity (Choi, Kim, Kim, Ku, & Sohn, 2014). Further, the FRe has synergic probiotic effects with yoghurt (Choi, Kim, Kim, Lee, Sohn, et al., 2014) and growth‐stimulating effects on lactic acid bacteria (i.e., *Bifidobacterium lactis, Lactobacillus acidophilus, and Streptococcus thermophilus*) responsible for gut health (Lee, Cho, Kim, Lim, et al., 2014). Given that the oligosaccharides and *Bificobacteria* themselves can promote the growth of lactic acid bacteria (Hopkins, Cummings, & Macfarlane, 1998), the dietary FRe may be effective in IBD patients. Thus, the therapeutic potential of FRe for UC was assessed in comparison with a treatment with sulfasalazine that down‐regulates nuclear factor‐kappa B (NF‐κB) pathway (Gan, Chen, & Ouyang, 2005).

## 2 MATERIALS AND METHODS

### 2.1 Preparation of FRe

FRe was prepared by the Glucan Corp., Ltd. and provided by NOVAREX. The rice was processed by triple fermentation: saccharification and fermentation with *Saccharomyces cerevisiae* (ATCC 9804; ATCC) and then *Weissella cibaria* (KACC 11845; KACC). It was lyophilized and stored at 4°C until use. The FRe yield was 36%. High‐performance liquid chromatography showed 73.02 mg/g galacto‐oligosaccharides (25.2 mg/g stachyose and 47.8 mg/g raffinose) as a main ingredient of FRe (Figure S1).

### 2.2 Animals

All animal experiments were performed according to the international regulations of the usage and welfare of laboratory animals and approved by the Institutional Animal Care and Use Committee at Daegu Haany University (Gyeongsan, Korea, Approval No. DHU2018‐068). Six‐week‐old male C57BL/6 mice were purchased from OrientBio, Inc.. They were maintained in a temperature (20–25°C) and humidity (50%–55%) controlled room with a 12‐hr light/12‐hr dark cycle under specific pathogen‐free condition. Food and water were supplied ad libitum. The animals were acclimatized for a week.

### 2.3 UC induction and treatment

A total of 48 mice were divided into six groups (*n* = 8/group) with similar body weights. The mice were allocated to individual metabolic cages (DJ‐271; Daejong) with food and water. For the UC model, five groups received drinking water with 3.5% dextran sodium sulfate (DSS; Sigma‐Aldrich), as described previously (Chen, Zhang, Dai, & Tang, 2018; Kim et al., 2013). One group, the intact control (intact), received water only. The drinking water was exchanged daily. One hour after the exchanges, the mice were orally administered in a volume of 10 ml/kg once a day for a week as follows: The intact and one group of the UC model (control) received distilled water, and the other four groups of UC model received sulfasalazine (Sigma‐Aldrich) at 100 mg/kg (sulfasal) or FRe at 300, 200, and 100 mg/kg (FRe300, FRe200, and FRe100, respectively). The animals were fasted overnight before the initial treatment and euthanasia using CO_2_, and the body weights were measured daily. They were monitored for diarrhea and stool condition a day after all treatments; the bloods and tissue samples of the cecum to the rectum were collected at euthanasia.

### 2.4 Disease activity index and colorectal weight and length

Disease activity index (DAI) was calculated as the total mean score for three parameters, body weight loss, stool condition, and rectal bleeding, as described previously (Kim et al., 2013). The parameters were scored as 0, 1, 2, 3, and 4 in the following order: for weight loss, <1%, 1%–5%, 5%–10%, 10%–15%, and >15%; for stool condition, normal solid pellets, soft but adherent pellet‐shape, loose stool with some solidity, loose stool with liquid consistency, and diarrhea; for rectal bleeding, negative, negative but visual signs in pellet, and slight, visual and gross signs in pellet and rectum, respectively. The sample of the cecum to the rectum was weighed and expressed as a percentage of body weight. Then, the colorectal length was measured.

### 2.5 Analysis of colonic microbiota

The colon contents were aseptically collected, diluted 10‐fold, and cultured in triplicate (Filipescu et al., 2018). For a culture of *E. coli* and *Enterococci*, Chromocult agar and Bile Esculin Azide Agar were incubated in aerobiosis at 37°C for 48 hr. For a culture of the anaerobes, *Lactobacillus* spp. and *Bifidobacterium* spp., Brain Heart Infusion agar, de Mann, Rogosa, and Sharpe (MRS) agar, modified MRS agar, 0.3% sodium propionate, 0.2% lithium chloride, 0.05% cysteine hydrochloride, and 5% defibrinated sheep blood were used. The cultures were incubated by the use of AnaeroJars and AnaeroGen sachets at 37°C for 72 hr. The colonies were measured as log colony‐forming unit (CFU)/g colon content. All of the agars and reagents were purchased from Becton Dickinson.

### 2.6 Assessment of serum immunoglobulin (Ig)

Blood collected via the inferior vena cava was centrifuged at 14,000 *g* for 10 min at 4°C, and the resultant serum was prepared. The levels of IgG, IgA, and IgM were assessed at 450 nm using enzyme‐linked immunosorbent assay (ELISA) kits, MBS720755, MBS702698, and MBS703424 (MyBioSource), respectively, according to the manufacturer's instructions.

### 2.7 Assessment of cytokine levels

Cytokine levels were examined in the serum and colon tissue. For the tissue levels, a portion of the colon was disintegrated in five volumes of ice‐cold RIPA buffer and incubated for 30 min on ice. The tissue supernatant was prepared by centrifuging at 20,000 *g* for 15 min at 4°C. The levels of TNF‐α, IL‐6, and IL‐8 were assessed at 450 nm using ELISA kits, MBS825075 (MyBioSource), MBS2508516 (MyBioSource), and DElA1355 (Creative Diagnostics), respectively. The total protein was measured by the Lowry method using a bovine serum albumin.

### 2.8 Myeloperoxidase activity assay

Myeloperoxidase (MPO) was extracted from the colonic homogenates in 10 volumes of ice‐cold 50 mM potassium phosphate buffer (pH 6.0) containing 0.5% hexadecyltrimethyl‐ammonium bromide (Sigma). The MPO activity was measured by the H_2_O_2_‐dependent oxidation of o‐dianizidine 2 HCl. One unit (U) was defined as the amount of the MPO/g tissue weight that caused a change in absorbance of 1.0/min at 460 nm.

### 2.9 Antioxidant defense system

For malondialdehyde (MDA), the colonic homogenates in 10 mM Tris‐HCl (pH 7.4) were centrifuged at 12,000 *g* for 15 min, and the supernatant was assessed by the thiobarbituric acid reactivity (Jamall & Smith, 1985). The value was expressed as nM/mg protein at 525 nm. For glutathione (GSH), the homogenates in 50 mM Tris‐HCl buffer containing 20 mM EDTA and 0.2 mM sucrose (pH 7.5) were precipitated with 25% trichloroacetic acid and centrifuged at 3,000 *g* for 15 min at 4ºC. The supernatant was treated with 2‐nitrobenzoic acid (Sigma‐Aldrich), and the reduced value in the samples against a blank was expressed as nM/mg tissue at 412 nm. The activities of catalase and superoxide dismutase (SOD) were assessed as described previously (Lim et al., 2019). One U of catalase activity was defined as the amount of the enzyme that was required to decompose H_2_O_2_ per min (mM min^−1^ mg^−1^ tissue protein) at 240 nm for 100 s. One U of SOD activity was defined as the SOD amount that diminished the initial absorbance of the nitroblue tetrazolium formazan by 50% at 540 nm (mM min^−1^ mg^−1^ tissue).

### 2.10 Real‐time reverse transcription polymerase chain reaction (RT‐PCR)

The colonic total RNA was extracted using TRIzol reagent (Invitrogen) and reverse‐transcribed using the reagent High‐Capacity cDNA Reverse Transcription Kit (Applied Biosystems). Reverse transcription polymerase chain reaction (RT–PCR) was performed in a CFX96^TM^ Real‐Time System (Bio‐Rad) and analyzed through the ABI Step–One–Plus Sequence Detection System (Applied Biosystems). The specific primers (Bioneer) are listed in Table S1. The relative expression was calculated through the 2^−ΔΔCt^ method. Glyceraldehyde‐3‐phosphate dehydrogenase (GAPDH) was used for normalization.

### 2.11 Histopathology

The colon sample taken at 1.5 cm apart from the cecum was fixed in 10% neutral buffered formalin and cross‐trimmed. It was paraffin‐embedded and serially sectioned at a thickness of 3–4 μm. The sections were stained with hematoxylin–eosin (HE) or Masson's trichrome (MT). The histomorphometric analyses were performed using an automated image analyzer (iSolution FL ver. 9.1, IMT i‐Solution Inc.). The mucosa damage was scored as 0, +1, +2, +3, and +4 for normal, loss of one‐third of the mucosa, loss of two‐thirds of the mucosa, lamina propria covered with a single‐layered epithelium with mild inflammation, and erosion with evident inflammation, respectively (Kim et al., 2013). The histopathologist was blinded to the treatment.

### 2.12 Immunohistochemistry

The other sections were pretreated with citrate buffer (pH 6.0) for antigen retrieval as previously described (Park, Ku, Lee, & Kim, 2012). The endogenous peroxidase was removed by treatment with 0.3% H_2_O_2_, and nonspecific protein was blocked by normal serum. The sections were incubated with primary antibodies overnight at 4ºC as follows: TNF‐α (sc‐52746, Santa Cruz Biotechnology; a dilution at 1:200), cluster of differentiation (CD)4 (sc‐7219, Santa Cruz Biotechnology; a dilution at 1:100), and CD8 (ab4055, Abcam; a dilution at 1:100). Then, they were incubated with a biotinylated‐secondary antibody and ABC reagents (Vector Labs) for 1 hr. The immunoreactivity was visualized using peroxidase substrate kit (Vector Labs) for 3 min. All of the sections were incubated in a humidity chamber and rinsed 3 times with 0.01 M phosphate‐buffered saline between each step. The cells occupying more than 20% of the immunoreactivities were regarded as positive. The histopathologist was blinded to the treatment.

### 2.13 Statistical analysis

The data were expressed as the mean ± standard deviation of the eight samples. First, the variance homogeneity was examined by Levene's test. If no significance was found, the data were analyzed by one‐way analysis of variance (ANOVA) followed by a least significant difference (LSD) post hoc test. When significance was found, a nonparametric comparison test, the Kruskal–Wallis H test, was performed, and this was followed by the Mann–Whitney *U* (MW) post hoc test with a Bonferroni correction. The kinetic body weight changes were examined by two‐way ANOVA with the main factors of the group and time point measured. The time point was treated as a repeated measure. A *p*‐value <.05 was considered significant.

## 3 RESULTS

### 3.1 Effects on UC symptoms

**Figure 1:**
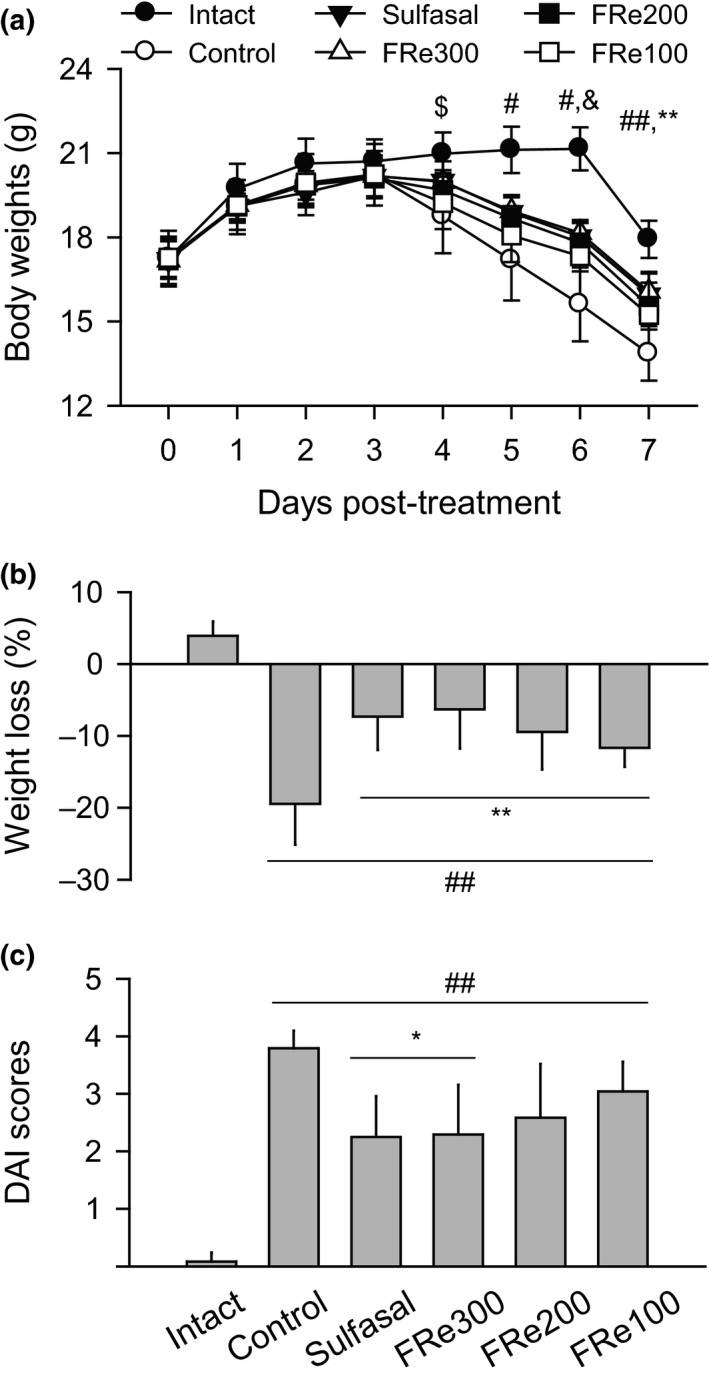
Body weight changes and disease activity index. Kinetic body weights and the weight loss on days 0 to 7 post‐treatment were shown in (a) and (b), respectively. Disease activity index (DAI) was examined after all treatments (c). Values are expressed as means ± standard deviation (*SD*) of eight samples. ^$^ *p* < .05 control versus intact, ^#^ *p* < .05 and ^##^ *p* < .01 versus intact, ^&^ *p* < .05 sulfasal, FRe300, and FRe200 versus control, ^*^ *p* < .05 and ^**^ *p* < .01 versus control

The two‐way ANOVA for the kinetic body weight changes showed significant differences in the groups (*F* = 9.8, *p* < .01) and significant interactions between the groups and the time point (*F* = 17.9, *p* < .01, Figure 1a). Comparing to the intact group, the kinetic changes were significantly decreased in the control group on days 4 to 7 post‐treatment and in the sulfasal and FRe groups on days 5 to 7 (*p* < .05). However, the changes versus the control group were significantly inhibited in the sulfasal, FRe300, and FRe200 groups on days 6 and 7 post‐treatment and in the FRe100 group on day 7 (*p* < .05). The ratios of the weight loss on days 0 to 7 post‐treatment were significantly lower in the sulfasal and FRe groups than in the control (*p* < .01, Figure 1b); the weight loss was 7.3%, 6.3%, 9.5%, and 11.7% in the sulfasal, FRe300, FRe200, and FRe100 groups, respectively, but 19.5% in the control. The DAI scores were still higher in all groups than the intact group after all treatments (*p* < .01, Figure 1c). However, the DAI scores and each parameter scores for weight loss, stool consistency, and rectal bleeding were significantly lower in the sulfasal and FRe300 groups than the control (*p* < .05). There were no significant changes in the scores of the FRe200 and FRe100 groups when compared with those of the control.

### 3.2 Effects on colorectal weight and length

**Figure 2:**
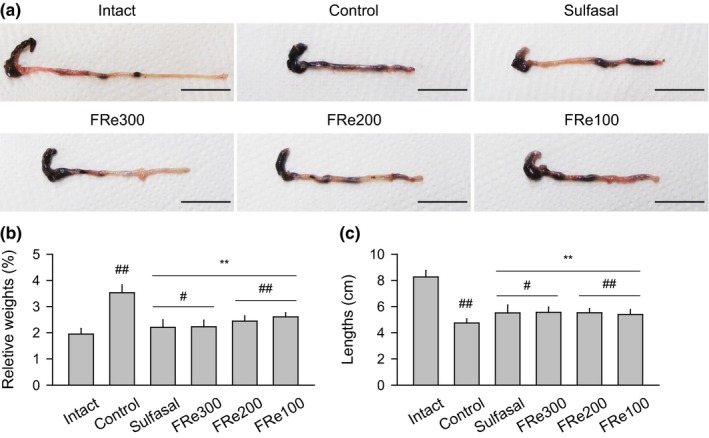
Colorectal weight and length. Representative samples of the cecum to the rectum are shown in (a). Scale bars = 2 cm. The relative organ weight to body weight (b) and the colorectal lengths (c) were expressed as means ± *SD* of eight samples. ^#^ *p* < .05 and ^##^ *p* < .01 versus intact, and ^**^ *p* < .01 versus control

The colon was observed to be swollen and erosive in the control after all the treatments; however, the lesions were mild in the sulfasal and FRe groups (Figure 2a). Comparing to the intact group, the control group showed significant increases in the relative weight of the cecum to the rectum and decreases in the colorectal length (*p* < .01, Figure 2b,c). However, the sulfasal and FRe groups showed increases in the relative weight and decreases in the length when compared with the control (*p* < .01).

### 3.3 Effects on colonic microbiota

**Figure 3:**
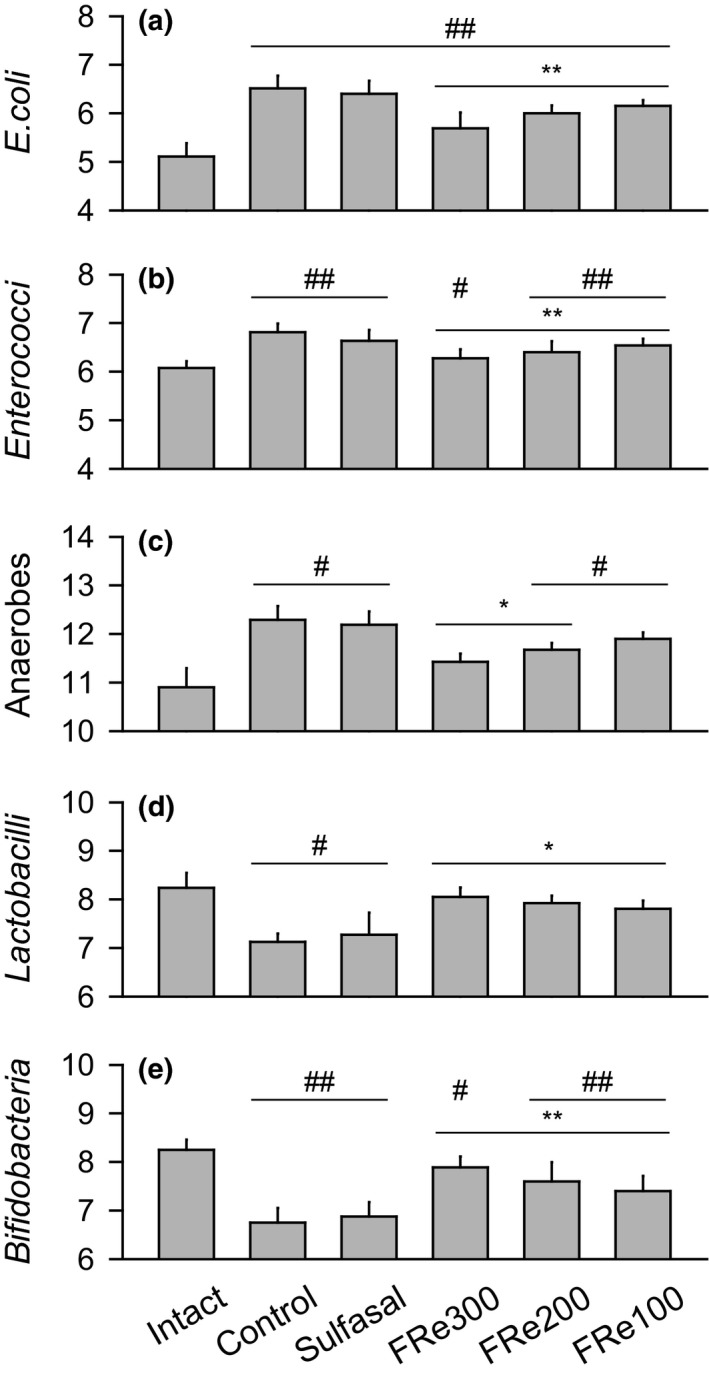
Colonic microbiota. Colony of *Echerichia (E.) coli*, *Enterococci*, anaerobes, *Lactobacilli*, and *Bifidobacteria* was counted and expressed as means ± *SD* (log colony‐forming unit/g of colon contents) of eight samples. ^#^ *p* < .05 and ^##^ *p* < .01 versus intact, and ^*^ *p* < .05 and ^**^ *p* < .01 versus control

The colonies of the cultured *E. coli* and *Enterococci* were more in all of UC model than the intact group; however, they were fewer in the FRe groups than the control (*p* < .05, Figure 3a,b). Similarly, the colony of anaerobes was more in the control than the intact, but it was fewer in the FRe300 and FRe200 groups than the control (*p* < .05, Figure 3c). Conversely, while the colonies of *Lactobacilli* and *Bifidobacteria* were fewer in the control than the intact, they were more in the FRe groups than the control (*p* < .05, Figure 3d,e). In particular, the colonies of anaerobes in the FRe300 and *Lactobacilli* in all of the FRe groups were not different from those in the intact. However, there were no differences in the microbiota colonies between the sulfasal and control groups.

### 3.4 Effects on antioxidant activity and immune responses

**Figure 4:**
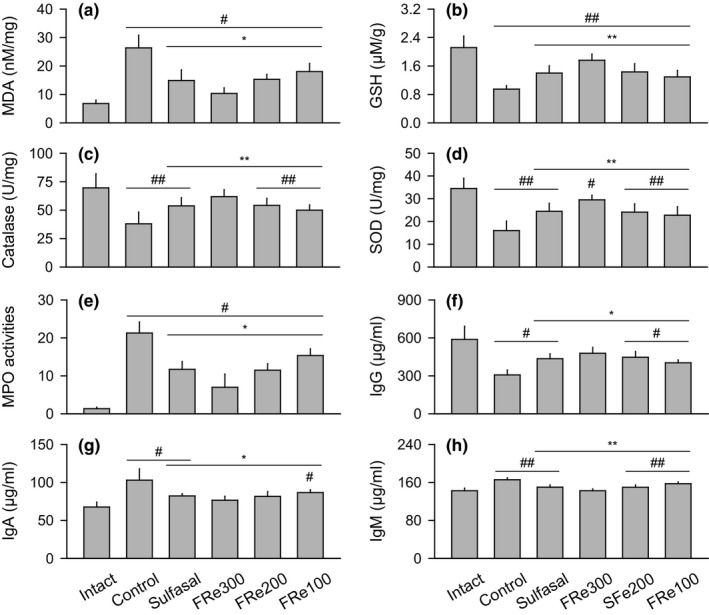
Colonic antioxidant activity and immune response. Colonic antioxidant activities were assessed by levels of malondialdehyde (MDA) and glutathione (GSH), and activities of catalase and superoxide dismutase (SOD) (a–d). The immune responses were assessed by tissue levels of myeloperoxidase (MPO; e) and serum immunoglobulins (Igs; f–h). Values were expressed as means ± *SD* of eight samples. ^#^ *p* < .05 and ^##^ *p* < .01 versus intact, and ^*^ *p* < .05 and ^**^ *p* < .01 versus control

Comparing to the intact group, the control group showed significant increases in the tissue levels of MDA, along with decreases in the levels of GSH and activities of catalase and SOD (*p* < .05, Figure 4a–d). However, the results were significantly reversed in the sulfasal and FRe groups compared with the control (*p* < .05). In addition, comparing to the intact group, the control group showed significant increases in the tissue levels of MPO activity and serum levels of IgA and IgM but decreases in the levels of IgG (*p* < .05, Figure 4e–h). However, comparing to the control group, the sulfasal and FRe groups showed significant decreases in the levels of MPO, IgA, and IgM and increases in the levels of IgG (*p* < .05).

### 3.5 Expressions of proinflammatory cytokines

**Figure 5:**
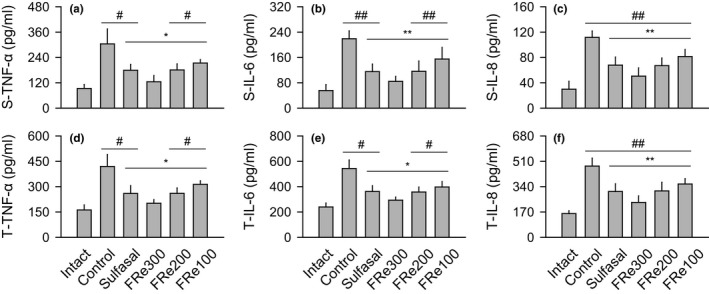
Production of proinflammatory cytokines. Serum (S) and tissue (T) levels of tumor necrosis factor (TNF)‐α, interleukin (IL)‐6, and IL‐8 were expressed as means ± *SD* of eight samples. ^#^ *p* < .05 and ^##^ *p* < .01 versus intact, and ^*^ *p* < .05 and ^**^ *p* < .01 versus control

**Figure 6:**
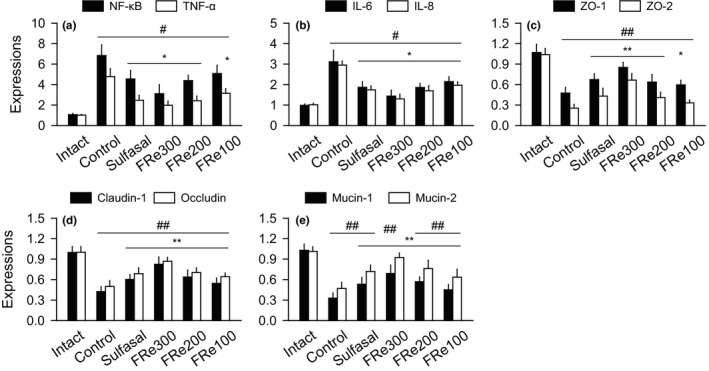
Gene expression involved in inflammation and intestinal barrier integrity. Gene expressions of nuclear factor‐kappa B (NF‐κB), TNF‐α, IL‐6, and IL‐8 were assessed for the inflammation severity (a and b). The expressions of zona occludens (ZO)‐1, ZO‐2, claudin, occludin, mucin‐1, and mucin‐2 were assessed for the intestinal barrier integrity (c–e). Values were expressed as means ± *SD* of eight samples. ^#^ *p* < .05 and ^##^ *p* < .01 versus intact, and ^*^ *p* < .05 and ^**^ *p* < .01 versus control

Similar tendencies were observed in both serum and colonic tissue levels of TNF‐α, IL6, and IL‐8 (Figure 5); the levels were significantly increased in the control versus the intact, but decreased in the sulfasal and FRe groups versus the control (*p* < .05). Consistently, their mRNA expressions were significantly up‐regulated in the control versus the intact, but down‐regulated in the sulfasal and FRe groups versus the control (*p* < .05, Figure 6a,b). The mRNA expression of NF‐κB was also significantly up‐regulated in the control versus the intact, but they were down‐regulated in the sulfasal, FRe300, and FRe200 groups versus the control (*p* < .05).

### 3.6 Gene expression involved in the intestinal barrier integrity

The expressions of mRNAs for zona occludens (ZO)‐1, ZO‐2, Claudin‐1, Occludin, Mucin‐1, and Mucin‐2 were significantly down‐regulated in the control group compared with the intact (*p* < .01, Figure 6c–e). Although there were no significances in the ZO‐2 expression of the FRe 100 group, the other expressions were significantly up‐regulated in the sulfasal and FRe groups compared with the control group (*p* < .05).

### 3.7 Histopathological and immunohistochemical analyses

**Figure 7:**
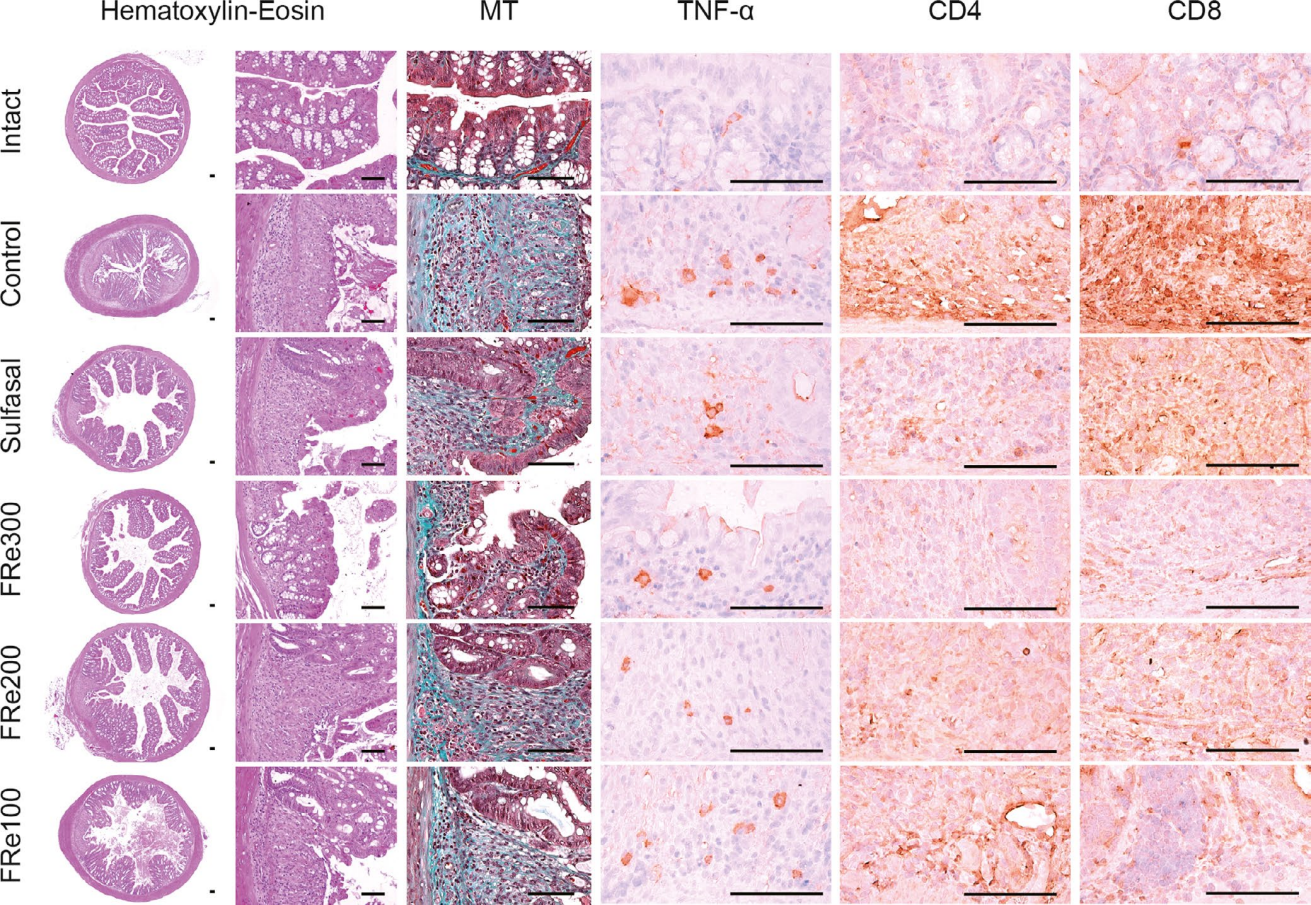
Histopathological and immunohistochemical analyses. The colonic tissue sections were stained with hematoxylin–eosin and Masson's trichrome (MT), and other serial sections were immunostained for TNF‐α, cluster of differentiation (CD)4 and CD8. Scale bars = 100 μm

The DSS‐induced colorectal lesions exhibited massive infiltration of the multifocal inflammatory cells, damaged crypts, epithelioglandular hyperplasia, submucosal erosions, and perimucosal edema and congestion in HE stain (Figure 7). The number of goblet cells was evidently reduced with the impaired mucosal integrity. Furthermore, the control group showed increased collagen‐deposited areas in MT stains and immunoreactivities for TNF‐α, CD4, and CD8. However, the histopathological lesions were mild in the FRe groups. The histomorphometric analyses revealed significant decreases in the villi height and width in the control versus the intact, but increases in the mucosal damage scores with collagen fiber areas and numbers of inflammatory cells (Table 1). However, it was reversed in the sulfasal and FRe groups compared with the control, excepting the inflammatory cells of the FRe100 group (*p* < .05). The immunoreactive cells for the TNF‐α, CD4, and CD8 as markers of inflammatory progress were more in the control than in the intact; however, they were significantly fewer in the sulfasal and FRe groups than in the control (*p* < .05). There were no differences in the total colonic diameter among the groups.

**Table 1:** Histomorphometric analyses on the colonic tissues of ulcerative colitis model

	Intact	Control	Sulfasalazine	FRe300	FRe200	FRe100
Mucosal damage	0.3 ± 0.5	3.6 ± 0.5^##^	2.1 ± 0.6^##,**^	1.3 ± 0.7^##,**^	2.0 ± 0.8^##,**^	2.4 ± 0.5^##,**^
Inflammatory cells	28.0 ± 10.1	325.3 ± 49.6^#^	212.4 ± 31.0^#,*^	157.3 ± 44.0^#,*^	220.5 ± 26.4^#,*^	257.3 ± 16.5^#^
Lumen diameters	2.2 ± 0.2	2.2 ± 0.2	2.2 ± 0.1	2.2 ± 0.2	2.2 ± 0.1	2.2 ± 0.2
Villus height	848.9 ± 43.9	202.2 ± 20.4^#^	628.8 ± 107.2^#,*^	689.0 ± 78.5^#,*^	629.8 ± 121.1^#,*^	466.0 ± 112.8^#,*^
Villus width	291.8 ± 27.9	172.1 ± 25.8^##^	240.8 ± 26.1^##,**^	262.2 ± 14.9^#,**^	238.6 ± 18.0^##,**^	209.6 ± 16.9^##,**^
Collagen fibers	18.9 ± 7.0	64.1 ± 7.0^##^	43.1 ± 4.4^##,**^	30.6 ± 5.9^##,**^	42.1 ± 5.4^##,**^	50.5 ± 10.6^##,**^
TNF‐α (+) cells	8.8 ± 4.0	76.3 ± 12.5^#^	26.8 ± 8.1^#,*^	15.0 ± 4.3^*^	27.0 ± 4.1^#,*^	42.3 ± 10.5^#,*^
CD4 (+) cells	8.5 ± 2.8	187.3 ± 26.6^#^	86.0 ± 32.0^#,*^	49.0 ± 19.4^#,*^	90.3 ± 27.1^#,*^	112.3 ± 27.9^#,*^
CD8 (+) cells	15.0 ± 3.6	247.9 ± 33.8^#^	124.5 ± 26.0^#,*^	61.3 ± 21.6^#,*^	127.0 ± 29.5^#,*^	188.6 ± 19.5^#,*^

Note

Histomorphometric analyses on stains in Figure 7 were expressed as means ± standard deviation of eight samples. Mucosal damage (scores), inflammatory cells (number/mm^2^), lumen diameters (mm), and villus height and width (μm) were assessed in hematoxylin–eosin stains, and collagen fibers (%/mm^2^) were assessed in Masson's trichrome stains. The immunopositive cells for tumor necrosis factor (TNF)‐α, cluster of differentiation (CD)4 and CD8 (number/mm^2^) were assessed in the corresponding immunostains. ^#^ *p* < .05 and ^##^ *p* < .01 versus intact, and ^*^ *p* < .05 and ^**^ *p* < .01 versus control.

## 4 DISCUSSION

The DSS‐induced model exhibited acute UC symptoms, such as weight loss, bloody stools, and colonic shortening, in agreement with other studies (Chen et al., 2018; Kim et al., 2013; Shen et al., 2019). However, the oral administration of FRe reduced the weight loss, DAI scores, and colon weight and increased the colorectal length. The histopathological analyses revealed mild colonic lesions with inhibited inflammatory progress in the FRe groups. Indeed, the FRe reduced neutrophil infiltration and production of the proinflammatory cytokines and up‐regulated the gene expressions responsible for the intestinal barrier integrity. Furthermore, the FRe enhanced antioxidant activities and reduced lipid peroxidation. Interestingly, the FRe treatments, especially for the FRe300 and FRe200 groups, attenuated the dysbiosis in the UC model despites no effects in the sulfasal group.

There are accumulating reports that elevated proinflammatory cytokines and the related chemokines are present in the colons of IBD patients (Monteleone, Vavassori, Biancone, Monteleone, & Pallone, 2002). The proinflammatory cytokines amplify the inflammatory cascade of the inflammatory mediators, destructive enzymes, and free radicals that cause tissue damage. NF‐κB is a transcription factor promoting the inflammation by the secretion of proinflammatory cytokines, activating production of a variety of inflammatory mediators (Spehlmann & Eckmann, 2009). Indeed, the sustained activation of NF‐κB has been involved in the inflammatory progress of IBDs including UC (Schreiber, Nikolaus, & Hampe, 1998). In this context, drugs suppressing NF‐κB activation can be a potential anti‐inflammatory strategy for IBDs (Park et al., 2012). The present results showed that the levels of TNF‐α, IL‐6, and IL‐8 were reduced in the FRe groups, which were accompanied by down‐regulation of NF‐κB, similar to those for the treatment group with sulfasalazine that inhibits NF‐κB pathway (Gan et al., 2005). The histopathological analyses showed consistent data; reduced collagen fibers, inflammatory cell infiltration, and mucosal damages in the colonic tissues of the FRe groups. It suggests NF‐κB pathway‐mediated anti‐inflammatory effects of FRe.

Proinflammatory cytokines are known to trigger the chemotactic accumulation of leukocytes, mainly neutrophil (McKnight et al., 1996). The increased neutrophil infiltration induces the overproduction of reactive oxygen species as well as the inflammation damage, and the excessive oxidative stress further aggravates the inflammation (Piechota‐Polanczyk & Fichna, 2014). Here, the DSS induction increased the MDA levels with the depletion of the colonic antioxidant state. However, the FRe reduced lipid peroxidation probably due to the enhanced antioxidant activities (i.e., GSH, catalase, and SOD) or reduced MPO‐derived oxidants. The antioxidant effects of FRe may correlatively inhibit the inflammatory progression and colonic tissue damages by maintaining oxidative stress at low levels (Malle, Furtmüller, Sattler, & Obinger, 2007).

The gut microbiota plays a key role in not only inhibiting colonization by pathogens, such as *E. coli*, *Enterococci*, and anaerobes, but also conferring mucosal barrier functions (Belkaid & Hand, 2014). The microbiota normally inhibits NF‐κB activation in the gut epithelial cells, which attribute the anti‐inflammatory and antioxidant properties as well (Cerf‐Bensussan & Gaboriau‐Routhiau, 2010). Our UC model exhibited the colonic dysbiosis similar with other studies in IBD patients (Matsuoka & Kanai, 2015) and UC animal model (Filipescu et al., 2018; Shen et al., 2019) increases in the colonies of *E. coli* and *Enterococci*, but decreases in *Lactobacilli* and *Bifidobacteria*. Some intestinal gram‐negative bacteria, particularly *E. coli*, have been characterized as involving activation of inflammation and oxidative stress and exacerbation of the mucosal injuries (Darnaud et al., 2018). Conversely, specific bacteria of *Bifidobacterium* species (i.e., *B. bifidum* S17 and *B. lactis*) and *Lactobacillus* species (i.e., *L. casei* BL12, *L. paracasei* B21060, and *L. fermentum* ACA‐DC 179) have shown anticolitis effects via anti‐inflammatory (Liu, Su, Ong, Cheng, & Tsai, 2011) and antimicrobial activity (Jeong et al., 2015). Here, the FRe reduced the pathogenic bacteria (*E. coli*, *Enterococci*, and anaerobes) and preserved the beneficial bacteria (*Lactobacilli* and *Bifidobacteria*). Our previous studies have demonstrated the laxative effects of FRe on constipation (Choi et al., 2014) and the growth‐stimulating effects on lactic acid bacteria (Lee, Cho, Kim, Sohn, & Choi, 2014). In addition, the FRe enhances the laxative activity of yoghurt (Choi, Kim, Kim, Lee, Sohn, et al., 2014). Although the mechanisms are unclear, galacto‐oligosaccharides contained in the FRe may act as prebiotics to benefit colon health probably by selective stimulation of the specific microbiota and production of short chain fatty acids (i.e., acetate and butyrate) as a primary energy source for the intestinal epithelium (Blaut & Clavel, 2007). The promoting effects of FRe on the specific microbiota may be applied as an attractive therapeutic strategy for IBD including UC.

The intestinal barrier, composed of the mucous layer, epithelial cells, and the intercellular tight junctions, contributes to preventing the bacterial invasion and providing a defense mechanism to the pathogens (Correa‐Oliveira, Fachi, Vieira, Sato, & Vinolo, 2016). Our UC model exhibited thin mucus layer with reduced goblet cells and the impaired barrier integrity, similarly with UC patients (Pullan et al., 1994; Seldenrijk et al., 1991). The depletion of the mucus layer and disruption of the epithelial tight junctions cause hyperpermeability of the gut microbiota, which exacerbate the microbiota‐induced inflammation by activating helper T (Th) and B cells (Fyderek et al., 2009). However, the FRe increased the number of goblet cell and up‐regulated the gene expressions for mucins (*Mucin‐1* and *Mucin‐2*) and tight junctions (*ZO‐1*, *ZO‐2*, *Claudin‐1*, and *Occludin*). It may contribute to inhibiting the gut dysbiosis and the corresponding immune responses. Indeed, the immunoreactive cells for TNF‐α, CD4, and CD8 were reduced in the FRe groups compared with the control. It is considered that the well‐preserved intestinal barrier by the FRe treatment may inhibit the responses of the CD4^+^ and CD8^+^ T cells to the antigen‐presenting cells (McKnight et al., 1996).

In UC, Th2‐mediated humoral immunity prevails in the gut mucosal inflammatory response, and the mucosal plasma cells produce amount of Igs (Kett, Rognum, & Brandtzaeg, 1987). High levels of anticommensal Igs, IgG, IgA, and IgM are found in the inflamed colonic mucosa and lumen in the human patients and DSS‐induced colitis model (Castro‐Dopico et al., 2019), but the levels are not significant in the serum (Lin et al., 2018). Nevertheless, our UC model showed significant decreases in the serum levels of IgG but increases in the levels of IgA and IgM, and the FRe treatment reversed the levels in our experimental condition. Although similar results have been reported in another study (Han et al., 2015), it is difficult to speculate how the peripheral immune response in blood reacts with the mucosal immunity of UC. Given that proliferation of T cells isolated from the DSS‐induced colitis mouse is inhibited in the pretreatment with IgG (Shintani et al., 1998), the increased serum IgG may reduce the mucosal T‐cell response. Otherwise, it might be just results after the migration of serum Igs‐secreting cells to the mucosal lesions to compensate the alteration of the commensal Igs for maintaining gut homeostasis in a phase of the acute UC; FRe may increase the serum IgG to counteract the invading bacteria but reduce the serum IgM and IgA to the decreased local antigenic stimulation. It drives future studies on the additional complex mechanisms behind the inflammatory cascade reaction.

The treatments of UC have been currently focused on symptom control, mucosal healing, and complication avoidance (Chen et al., 2018). The available drugs require patients to receive long‐life treatment; however, they are not fully curative despite an enormous economic burden. Inexpensive nontoxic fermented rice is therefore one of the promising therapeutic approaches for IBD, including UC. The present study demonstrated for the first time the potent ameliorating effect of the oral administration of FRe on UC. This study provides useful information for the human gut health as a functional food and an important source of nutraceuticals.

## CONFLICT OF INTEREST

The authors declare that there are no conflicts of interest.

## ETHICAL APPROVAL

Ethical review: This study was approved by the Institutional Animal Care and Use Committee of Daegu Haany University (Gyeongsan, Korea, Approval No. DHU2018‐068).

## INFORMED CONSENT

Written informed consent was obtained from all study participants.

## References

[fsn31460-bib-0001] Asakura, H. , Suzuki, K. , Kitahora, T. , & Morizane, T. (2008). Is there a link between food and intestinal microbes and the occurrence of Crohn's disease and ulcerative colitis? Journal of Gastroenterology and Hepatology, 23(12), 1794–1801. 10.1111/j.1440-1746.2008.05681.x 19120872

[fsn31460-bib-0002] Belkaid, Y. , & Hand, T. W. (2014). Role of the microbiota in immunity and inflammation. Cell, 157(1), 121–141. 10.1016/j.cell.2014.03.011 24679531 PMC4056765

[fsn31460-bib-0003] Blaut, M. , & Clavel, T. (2007). Metabolic diversity of the intestinal microbiota: Implications for health and disease. Journal of Nutrition, 137(3 Suppl 2), 751S–755S. 10.1093/jn/137.3.751S 17311972

[fsn31460-bib-0004] Castro‐Dopico, T. , Dennison, T. W. , Ferdinand, J. R. , Mathews, R. J. , Fleming, A. , Clift, D. , … Clatworthy, M. R. (2019). Anti‐commensal IgG Drives Intestinal Inflammation and Type 17 Immunity in Ulcerative Colitis. Immunity, 50(4), 1099–1114 e1010. 10.1016/j.immuni.2019.02.006 30876876 PMC6477154

[fsn31460-bib-0005] Cerf‐Bensussan, N. , & Gaboriau‐Routhiau, V. (2010). The immune system and the gut microbiota: Friends or foes? Nature Reviews Immunology, 10(10), 735–744. 10.1038/nri2850 20865020

[fsn31460-bib-0006] Chassaing, B. , & Darfeuille‐Michaud, A. (2011). The commensal microbiota and enteropathogens in the pathogenesis of inflammatory bowel diseases. Gastroenterology, 140(6), 1720–1728. 10.1053/j.gastro.2011.01.054 21530738

[fsn31460-bib-0007] Chen, Y. L. , Zhang, Y. L. , Dai, Y. C. , & Tang, Z. P. (2018). Systems pharmacology approach reveals the antiinflammatory effects of *Ampelopsis grossedentata* on dextran sodium sulfate‐induced colitis. World Journal of Gastroenterology, 24(13), 1398–1409. 10.3748/wjg.v24.i13.1398 29632421 PMC5889820

[fsn31460-bib-0008] Choi, J. S. , Kim, J. W. , Cho, H. R. , Kim, K. Y. , Lee, J. K. , Ku, S. K. , & Sohn, J. H. (2014). Laxative effects of fermented rice extract (FRe) in normal rats. Toxicology and Environmental Health Sciences, 6(3), 155–163. 10.1007/s13530-014-0200-2

[fsn31460-bib-0009] Choi, J. S. , Kim, J. W. , Cho, H. R. , Kim, K. Y. , Lee, J. K. , Sohn, J. H. , & Ku, S. K. (2014). Laxative effects of fermented rice extract in rats with loperamide‐induced constipation. Experimental and Therapeutic Medicine, 8(6), 1847–1854. 10.3892/etm.2014.2030 25371743 PMC4218700

[fsn31460-bib-0010] Choi, J. S. , Kim, J. W. , Kim, K. Y. , Ku, S. K. , & Sohn, J. H. (2014). Single‐dose oral toxicity of fermented rice extracts (FREs): A 14‐day observation. Pakistan Journal of Pharmaceutical Sciences, 27(1), 129–137. 24374435

[fsn31460-bib-0011] Choi, J. S. , Kim, J. W. , Kim, K. Y. , Lee, J. K. , Sohn, J. H. , & Ku, S. K. (2014). Synergistic effect of fermented rice extracts on the probiotic and laxative properties of yoghurt in rats with loperamide‐induced constipation. Evidence‐Based Complementary and Alternative Medicine, 2014, 878503 10.1155/2014/878503 25214876 PMC4158107

[fsn31460-bib-0012] Correa‐Oliveira, R. , Fachi, J. L. , Vieira, A. , Sato, F. T. , & Vinolo, M. A. (2016). Regulation of immune cell function by short‐chain fatty acids. Clinical & Translational Immunology, 5(4), e73 10.1038/cti.2016.17 27195116 PMC4855267

[fsn31460-bib-0013] Darnaud, M. , Dos Santos, A. , Gonzalez, P. , Augui, S. , Lacoste, C. , Desterke, C. , … Faivre, J. (2018). Enteric delivery of regenerating family member 3 alpha alters the intestinal microbiota and controls inflammation in mice with colitis. Gastroenterology, 154(4), 1009–1023 e1014. 10.1053/j.gastro.2017.11.003 29133078

[fsn31460-bib-0014] Filipescu, I. E. , Leonardi, L. , Menchetti, L. , Guelfi, G. , Traina, G. , Casagrande‐Proietti, P. , … Brecchia, G. (2018). Preventive effects of bovine colostrum supplementation in TNBS‐induced colitis in mice. PLoS ONE, 13(8), e0202929 10.1371/journal.pone.0202929 30138385 PMC6107273

[fsn31460-bib-0015] Fiocchi, C. (1998). Inflammatory bowel disease: Etiology and pathogenesis. Gastroenterology, 115(1), 182–205. 10.1016/S0016-5085(98)70381-6 9649475

[fsn31460-bib-0016] Fyderek, K. , Strus, M. , Kowalska‐Duplaga, K. , Gosiewski, T. , Wedrychowicz, A. , Jedynak‐Wasowicz, U. , … Heczko, P. B. (2009). Mucosal bacterial microflora and mucus layer thickness in adolescents with inflammatory bowel disease. World Journal of Gastroenterology, 15(42), 5287–5294. 19908336 10.3748/wjg.15.5287 PMC2776855

[fsn31460-bib-0017] Gan, H. T. , Chen, Y. Q. , & Ouyang, Q. (2005). Sulfasalazine inhibits activation of nuclear factor‐kappaB in patients with ulcerative colitis. Journal of Gastroenterology and Hepatology, 20(7), 1016–1024. 10.1111/j.1440-1746.2005.03862.x 15955209

[fsn31460-bib-0018] Han, F. , Zhang, H. , Xia, X. , Xiong, H. , Song, D. , Zong, X. , & Wang, Y. (2015). Porcine β‐defensin 2 attenuates inflammation and mucosal lesions in dextran sodium sulfate–induced colitis. The Journal of Immunology, 194(4), 1882–1893. 10.4049/jimmunol.1402300 25601921

[fsn31460-bib-0019] Hanauer, S. B. (2006). Inflammatory bowel disease: Epidemiology, pathogenesis, and therapeutic opportunities. Inflammatory Bowel Diseases, 12(Suppl 1), S3–S9. 10.1097/01.MIB.0000195385.19268.68 16378007

[fsn31460-bib-0020] Ho, B. Y. , Wu, Y. M. , Hsu, Y. W. , Hsu, L. C. , Kuo, Y. H. , Chang, K. J. , & Pan, T. M. (2010). Effects of Monascus‐fermented rice extract on malignant cell‐associated neovascularization and intravasation determined using the chicken embryo chorioallantoic membrane model. Integrative Cancer Therapies, 9(2), 204–212. 10.1177/1534735410365079 20356949

[fsn31460-bib-0021] Hopkins, M. , Cummings, J. , & Macfarlane, G. (1998). Inter‐species differences in maximum specific growth rates and cell yields of bifidobacteria cultured on oligosaccharides and other simple carbohydrate sources. Journal of Applied Microbiology, 85(2), 381–386. 10.1046/j.1365-2672.1998.00524.x

[fsn31460-bib-0022] Jamall, I. S. , & Smith, J. C. (1985). Effects of cadmium on glutathione peroxidase, superoxide dismutase, and lipid peroxidation in the rat heart: A possible mechanism of cadmium cardiotoxicity. Toxicology and Applied Pharmacology, 80(1), 33–42. 10.1016/0041-008X(85)90098-5 4024106

[fsn31460-bib-0023] Jeong, J. J. , Kim, K. A. , Jang, S. E. , Woo, J. Y. , Han, M. J. , & Kim, D. H. (2015). Orally administrated Lactobacillus pentosus var. plantarum C29 ameliorates age‐dependent colitis by inhibiting the nuclear factor‐kappa B signaling pathway via the regulation of lipopolysaccharide production by gut microbiota. PLoS ONE, 10(2), e0116533 10.1371/journal.pone.0116533 25689583 PMC4331539

[fsn31460-bib-0024] Kett, K. , Rognum, T. O. , & Brandtzaeg, P. (1987). Mucosal subclass distribution of immunoglobulin G‐producing cells is different in ulcerative colitis and Crohn's disease of the colon. Gastroenterology, 93(5), 919–924. 10.1016/0016-5085(87)90552-X 3308623

[fsn31460-bib-0025] Kim, D. S. , Ko, J. H. , Jeon, Y. D. , Han, Y. H. , Kim, H. J. , Poudel, A. , … Hong, S. H. (2013). Ixeris dentata NAKAI reduces clinical score and HIF‐1 expression in experimental colitis in mice. Evidence‐Based Complementary and Alternative Medicine: Ecam, 2013, 671281 10.1155/2013/671281 24194783 PMC3782128

[fsn31460-bib-0026] Kim, K. M. , Yu, K. W. , Kang, D. H. , & Suh, H. J. (2002). Anti‐stress and anti‐fatigue effect of fermented rice bran. Phytotherapy Research, 16(7), 700–702. 10.1002/ptr.1019 12410560

[fsn31460-bib-0027] Komiyama, Y. , Andoh, A. , Fujiwara, D. , Ohmae, H. , Araki, Y. , Fujiyama, Y. , … Kanauchi, O. (2011). New prebiotics from rice bran ameliorate inflammation in murine colitis models through the modulation of intestinal homeostasis and the mucosal immune system. Scandinavian Journal of Gastroenterology, 46(1), 40–52. 10.3109/00365521.2010.513062 20735154

[fsn31460-bib-0028] Laurell, A. , & Sjoberg, K. (2017). Prebiotics and synbiotics in ulcerative colitis. Scandinavian Journal of Gastroenterology, 52(4), 477–485. 10.1080/00365521.2016.1263680 27931127

[fsn31460-bib-0029] Lee, J. K. , Cho, H.‐R. , Kim, K.‐Y. , Lim, J. M. , Jung, G. W. , Sohn, J. H. , & Choi, J.‐S. (2014). The growth‐stimulating effects of fermented rice extract (FRe) on lactic acid bacteria and *Bifidobacterium* spp. Food Science and Technology Research, 20(2), 479–483. 10.3136/fstr.20.479

[fsn31460-bib-0030] Lee, J. , Cho, H. , Kim, K. , Sohn, J. , & Choi, J. (2014). Lactic acid bacteria and *Bifidobacterium* spp. growth stimulating effects of fermented rice extract (FRe). Food Science and Technology Research, 20, 479–483.

[fsn31460-bib-0031] Lim, J.‐M. , Song, C.‐H. , Park, S.‐J. , Park, D.‐C. , Jung, G.‐W. , Cho, H.‐R. , … Choi, J.‐S. (2019). Protective effects of triple fermented barley extract (FBe) on indomethacin‐induced gastric mucosal damage in rats. BMC Complementary and Alternative Medicine, 19(1), 49 10.1186/s12906-019-2457-0 30786935 PMC6383278

[fsn31460-bib-0032] Lin, R. , Chen, H. , Shu, W. , Sun, M. , Fang, L. , Shi, Y. , … Liu, Z. (2018). Clinical significance of soluble immunoglobulins A and G and their coated bacteria in feces of patients with inflammatory bowel disease. Journal of Translational Medicine, 16(1), 359 10.1186/s12967-018-1723-0 30558634 PMC6296095

[fsn31460-bib-0033] Linares, D. M. , Gomez, C. , Renes, E. , Fresno, J. M. , Tornadijo, M. E. , Ross, R. P. , & Stanton, C. (2017). Lactic acid bacteria and bifidobacteria with potential to design natural biofunctional health‐promoting dairy foods. Frontiers in Microbiology, 8, 846 10.3389/fmicb.2017.00846 28572792 PMC5435742

[fsn31460-bib-0034] Liu, Y. W. , Su, Y. W. , Ong, W. K. , Cheng, T. H. , & Tsai, Y. C. (2011). Oral administration of Lactobacillus plantarum K68 ameliorates DSS‐induced ulcerative colitis in BALB/c mice via the anti‐inflammatory and immunomodulatory activities. International Immunopharmacology, 11(12), 2159–2166. 10.1016/j.intimp.2011.09.013 21996541

[fsn31460-bib-0035] Lu, H. E. , Jian, C. H. , Chen, S. F. , Chen, T. M. , Lee, S. T. , Chang, C. S. , & Weng, C. F. (2010). Hypoglycaemic effects of fermented mycelium of *Paecilomyces farinosus* (G30801) on high‐fat fed rats with streptozotocin‐induced diabetes. Indian Journal of Medical Research, 131, 696–701. 20516543

[fsn31460-bib-0036] Malle, E. , Furtmüller, P. , Sattler, W. , & Obinger, C. (2007). Myeloperoxidase: A target for new drug development? British Journal of Pharmacology, 152(6), 838–854. 10.1038/sj.bjp.0707358 17592500 PMC2078229

[fsn31460-bib-0037] Matsuoka, K. , & Kanai, T. (2015). The gut microbiota and inflammatory bowel disease. Seminars in Immunopathology, 37(1), 47–55. 10.1007/s00281-014-0454-4 25420450 PMC4281375

[fsn31460-bib-0038] McKnight, A. J. , Macfarlane, A. J. , Dri, P. , Turley, L. , Willis, A. C. , & Gordon, S. (1996). Molecular cloning of F4/80, a murine macrophage‐restricted cell surface glycoprotein with homology to the G‐protein‐linked transmembrane 7 hormone receptor family. Journal of Biological Chemistry, 271(1), 486–489. 10.1074/jbc.271.1.486 8550607

[fsn31460-bib-0039] Monteleone, I. , Vavassori, P. , Biancone, L. , Monteleone, G. , & Pallone, F. (2002). Immunoregulation in the gut: Success and failures in human disease. Gut, 50(Suppl 3), III60–III64. 10.1136/gut.50.suppl_3.iii60 11953335 PMC1867679

[fsn31460-bib-0040] Park, S. Y. , Ku, S. K. , Lee, E. S. , & Kim, J. A. (2012). 1,3‐Diphenylpropenone ameliorates TNBS‐induced rat colitis through suppression of NF‐kappaB activation and IL‐8 induction. Chemico‐Biological Interactions, 196(1–2), 39–49. 10.1016/j.cbi.2012.02.002 22410118

[fsn31460-bib-0041] Piechota‐Polanczyk, A. , & Fichna, J. (2014). Review article: The role of oxidative stress in pathogenesis and treatment of inflammatory bowel diseases. Naunyn‐Schmiedeberg's Archives of Pharmacology, 387(7), 605–620. 10.1007/s00210-014-0985-1 PMC4065336 24798211

[fsn31460-bib-0042] Pullan, R. D. , Thomas, G. A. , Rhodes, M. , Newcombe, R. G. , Williams, G. T. , Allen, A. , & Rhodes, J. (1994). Thickness of adherent mucus gel on colonic mucosa in humans and its relevance to colitis. Gut, 35(3), 353–359. 10.1136/gut.35.3.353 8150346 PMC1374589

[fsn31460-bib-0043] Ruemmele, F. M. (2016). Role of diet in inflammatory bowel disease. Annals of Nutrition & Metabolism, 68(Suppl 1), 33–41. 10.1159/000445392 27355913

[fsn31460-bib-0044] Sartor, R. B. (2008). Microbial influences in inflammatory bowel diseases. Gastroenterology, 134(2), 577–594. 10.1053/j.gastro.2007.11.059 18242222

[fsn31460-bib-0045] Schreiber, S. , Nikolaus, S. , & Hampe, J. (1998). Activation of nuclear factor κB in inflammatory bowel disease. Gut, 42(4), 477–484. 9616307 10.1136/gut.42.4.477 PMC1727068

[fsn31460-bib-0046] Seldenrijk, C. A. , Morson, B. C. , Meuwissen, S. G. , Schipper, N. W. , Lindeman, J. , & Meijer, C. J. (1991). Histopathological evaluation of colonic mucosal biopsy specimens in chronic inflammatory bowel disease: Diagnostic implications. Gut, 32(12), 1514–1520. 10.1136/gut.32.12.1514 1773958 PMC1379253

[fsn31460-bib-0047] Shen, P. , Zhang, Z. , Zhu, K. , Cao, H. , Liu, J. , Lu, X. , … Zhang, N. (2019). Evodiamine prevents dextran sulfate sodium‐induced murine experimental colitis via the regulation of NF‐κB and NLRP3 inflammasome. Biomedicine & Pharmacotherapy, 110, 786–795. 10.1016/j.biopha.2018.12.033 30554117

[fsn31460-bib-0048] Shintani, N. , Nakajima, T. , Okamoto, T. , Kondo, T. , Nakamura, N. , & Mayumi, T. (1998). Involvement of CD4^+^ T cells in the development of dextran sulfate sodium‐induced experimental colitis and suppressive effect of IgG on their action. General Pharmacology, 31(3), 477–481. 10.1016/S0306-3623(98)00004-4 9703223

[fsn31460-bib-0049] Spehlmann, M. E. , & Eckmann, L. (2009). Nuclear factor‐kappa B in intestinal protection and destruction. Current Opinion in Gastroenterology, 25(2), 92–99. 10.1097/MOG.0b013e328324f857 19528876

[fsn31460-bib-0050] Vanderhoof, J. A. , & Young, R. J. (1998). Use of probiotics in childhood gastrointestinal disorders. Journal of Pediatric Gastroenterology and Nutrition, 27(3), 323–332. 10.1097/00005176-199809000-00011 9740206

