# Supplementary material for: Protective effects of fermented rice extract on ulcerative colitis induced by dextran sodium sulfate in mice
Source: Food Sci Nutr. 2020 Feb 14;8(3):1718–28. doi: 10.1002/fsn3.1460 (PMC7063356; doi:10.1002/fsn3.1460)

Table S1. Specific primers used for quantitative RT-PCR.

| Target | Sequence (5’ – 3’) | GenBank No. |
| --- | --- | --- |
| NF-κB | F: CAATGGCTACACAGGACCA  R: CACTGTCACCTGGAACCAGA | NM_008689 |
| TNF-α | F: GCATGGTGGTGGTTGTTTCTGACGAT  R: GCTTCTGTTGGACACCTGGAGACA | NM_010851.2 |
| IL-6 | F: GAGTCACAGAAGGAGTGGCTAAGGA  R: CGCACTAGGTTTGCCGAGTAGATCT | NM_031168.1 |
| IL-8 | F: TCTCGGTGTAGAGCAAGG  R: TTCCCAAGTGCTGGTATT | NM_011339.2 |
| ZO-1 | F: TCATCCCAAATAAGAACAGAGC  R: GAAGAACAACCCTTTCATAAGC | XM_006540786.1 |
| ZO-2 | F: GCTTTGGTGTGGACCAAGAT  R: TCCATTATGGGTTTGCATGA | XM_006526909.1 |
| Claudin-1 | F: GCTGGGTTTCATCCTGGCTTCT  R: CCTGAGCGGTCACGATGTTGTC | NM_016674.4 |
| Occludin | F: AAGCAAGTGAAGGGATCTGC  R: GGGGTTATGGTCCAAAGTCA | NM_001205255.1 |
| Mucin-1 | F: TGGATTGTTTCTGCAGATTTT  R: CCTGACCTGAACTTGATGCT | NM_013605.2 |
| Mucin-2 | F: CCCAGAAGGGACTGTGTATG  R: TGCAGACACACTGCTCACA | NM_023566.3 |
| GAPDH | F: CAACGGCACAGTCAAGGCTGAGA  R: CTCAGCACCAGCATCACCCCAT | NM_017008.3 |

F: forward, GAPDH = glyceraldehyde-3-phosphate dehydrogenase, IL = interleukin, NF-κB = nuclear factor kappa B, R: reverse, RT-PCR = reverse transcription polymerase chain reaction, TNF = tumor necrosis factor, ZO = Zonna occludens

Fig. S1. High-performance liquid chromatography for FRe. High-performance liquid chromatography was performed for standard compounds (A) and for FRe (B).


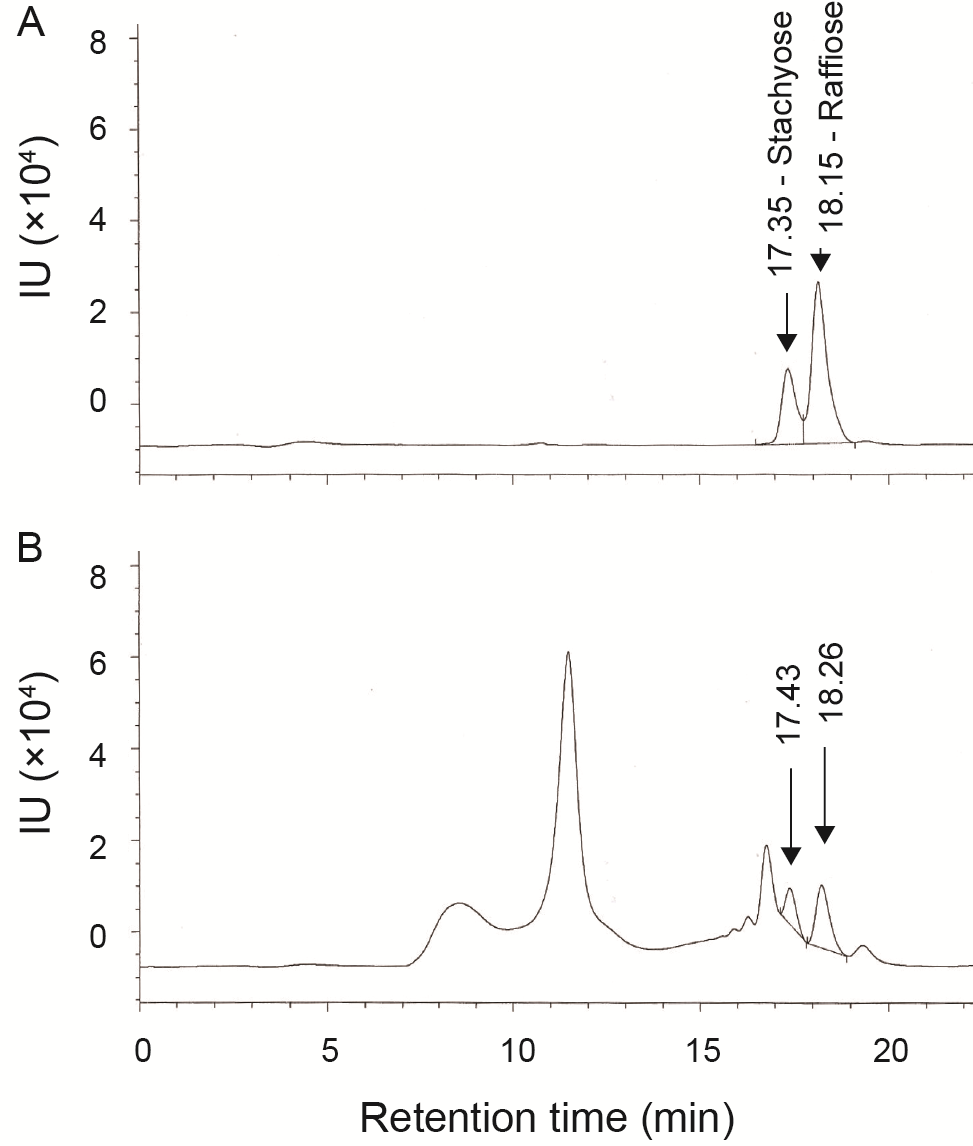

Supplement: Supplementary file 1 [file FSN3-8-1718-s001.docx]
